# Aortic haemodynamics: the effects of habitual endurance exercise, age and muscle sympathetic vasomotor outflow in healthy men

**DOI:** 10.1007/s00421-021-04883-2

**Published:** 2022-01-16

**Authors:** Denis J. Wakeham, Tony G. Dawkins, Rachel N. Lord, Jack S. Talbot, Freya M. Lodge, Bryony A. Curry, Lydia L. Simpson, Christopher J. A. Pugh, Robert E. Shave, Jonathan P. Moore

**Affiliations:** 1 Cardiff School of Sport and Health Sciences, Cardiff Metropolitan University, Cardiff, UK; 2 Cardiff and Vale University Health Board, University Hospital of Wales, Cardiff, UK; 3 School of Human and Behavioural Sciences, Bangor University, Bangor, UK; 4 Department of Sport Science, University of Innsbruck, Innsbruck, Austria; 5 Centre for Heart, Lung, and Vascular Health, University of British Columbia Okanagan, Kelowna, Canada

**Keywords:** Aortic haemodynamics, Habitual exercise, Age, Sympathetic vasomotor outflow

## Abstract

**Purpose:**

We determined the effect of habitual endurance exercise and age on aortic pulse wave velocity (aPWV), augmentation pressure (AP) and systolic blood pressure (aSBP), with statistical adjustments of aPWV and AP for heart rate and aortic mean arterial pressure, when appropriate. Furthermore, we assessed whether muscle sympathetic nerve activity (MSNA) correlates with AP in young and middle-aged men.

**Methods:**

Aortic PWV, AP, aortic blood pressure (applanation tonometry; SphygmoCor) and MSNA (peroneal microneurography) were recorded in 46 normotensive men who were either young or middle-aged and endurance-trained runners or recreationally active nonrunners (10 nonrunners and 13 runners within each age-group). Between-group differences and relationships between variables were assessed via ANOVA/ANCOVA and Pearson product-moment correlation coefficients, respectively.

**Results:**

Adjusted aPWV and adjusted AP were similar between runners and nonrunners in both age groups (all, *P* > 0.05), but higher with age (all, *P* < 0.001), with a greater effect size for the age-related difference in AP in runners (Hedges’ *g*, 3.6 vs 2.6). aSBP was lower in young (*P* = 0.009; *g* = 2.6), but not middle-aged (*P* = 0.341; *g* = 1.1), runners compared to nonrunners. MSNA burst frequency did not correlate with AP in either age group (young: *r* = 0.00, *P* = 0.994; middle-aged: *r* = − 0.11, *P* = 0.604).

**Conclusion:**

There is an age-dependent effect of habitual exercise on aortic haemodynamics, with lower aSBP in young runners compared to nonrunners only. Statistical adjustment of aPWV and AP markedly influenced the outcomes of this study, highlighting the importance of performing these analyses. Further, peripheral sympathetic vasomotor outflow and AP were not correlated in young or middle-aged normotensive men.

## Introduction

Aortic augmentation pressure (AP) represents a manifestation that presents in mid-systole due to the interaction between forward and backward travelling pressure waves, aortic stiffness (i.e. aortic pulse wave velocity, aPWV), and aortic reservoir pressure (O'Rourke and Mancia 1999; Mynard et al. 2020). Notably, AP and aPWV increase with age (McEniery et al. 2005) and contribute to the increase aortic systolic blood pressure (aSBP), which is predictive of future cardiovascular events in the general population (Lamarche et al. 2020). Whether lifestyle factors, such as habitual endurance exercise, influence AP, aPWV, and aSBP similarly is currently unclear.

As recently highlighted by Van Bortel et al. (2020), erroneous conclusions can be made when comparing aortic stiffness between individuals without the appropriate statistical adjustment of aPWV for mean arterial pressure (MAP). Furthermore, it is well known that habitual endurance exercise lowers heart rate (Katona et al. 1982) and that heart rate influences both AP and aPWV (Wilkinson et al. 2000, 2002; Tan et al. 2016). Therefore, in the presence of between-group differences in MAP and/or heart rate, statistical adjustments for these confounders should be made (Stoner et al. 2014). In the studies conducted to date comparing aPWV between endurance-trained and untrained individuals, the majority have not adjusted for MAP and/or heart rate when necessary and have reported lower aPWV in well-trained individuals (Vaitkevicius et al. 1993; McDonnell et al. 2013; Tarumi et al. 2013; Pierce et al. 2013, 2016). However, Shibata et al. (2018) reported no effects of lifelong exercise “dose” on aortic stiffness in middle-aged/older individuals. Unlike aPWV, AP, or more commonly reported augmentation index (AIx), is always adjusted for heart rate and is found to be lower in well-trained individuals (Vaitkevicius et al. 1993; McDonnell et al. 2013; Tarumi et al. 2013; Pierce et al. 2013, 2016; Shibata et al. 2018). Furthermore, not all studies include height or MAP (Stoner et al. 2014) as covariates when comparing AP or AIx between groups. Thus, to the best of our knowledge, no study has reported on the effects of habitual endurance exercise on aPWV and AP, with appropriate statistical adjustments for potential confounders.

**Fig. 1:**
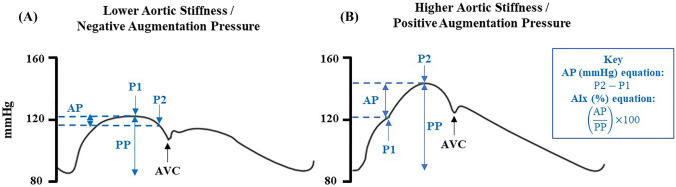
Effects of aortic stiffness and augmentation pressure on the ascending aortic blood pressure waveform. **A**, **B** These panels represent the effects of differences in aortic stiffness and augmentation pressure on aortic blood pressure and systolic pressure augmentation. Augmentation pressure (AP) and index (AIx) quantify systolic pressure augmentation; the equations to calculate AP and AIx are shown in the box within (**B**). Pressure 1 (P1) is taken as aortic systolic blood pressure (aSBP) in (**A**), whereas pressure 2 (P2) is recorded as aSBP in (**B**), due to positive systolic pressure augmentation in this instance. Aortic diastolic pressure is determined from the nadir of the waveform immediately prior to ventricular ejection which leads to the upstroke of the pressure waveform. Aortic valve closure (AVC) is noted at the incisura, which represents the end of ventricular ejection**.** *AIx *augmentation index, *AP *augmentation pressure, *aPP *aortic pulse pressure,* AVC *aortic valve closure,* P1 *pressure 1, *P2 *pressure 2

As well as habitual exercise, sympathetic vasomotor outflow to the skeletal muscle (i.e. muscle sympathetic nerve activity [MSNA]) could also influence aortic haemodynamics. MSNA is well known to play a pivotal role in the dynamic beat-by-beat control of peripheral vascular tone, and therefore peripheral blood pressure (Charkoudian and Wallin 2014; Shoemaker et al. 2015; Dampney 2017). However, less is known about whether MSNA also influences aortic haemodynamics. Previously, basal MSNA burst frequency and incidence have been shown to positively correlate with AP in normotensive young men (Casey et al. 2011; Smith et al. 2015), postmenopausal women with elevated blood pressure (i.e. [pre]hypertension) (Hart et al. 2013) and normotensive heart failure patients with reduced ejection fraction (Millar et al. 2019). In contrast, MSNA and AP were not correlated in a mixed-sex sample of healthy normotensive middle-aged individuals (Millar et al. 2019). Casey et al. (2011) suggested that MSNA likely influences AP via alterations in peripheral vascular tone, and subsequent increases in the magnitude of backward travelling pressure waves, as well as increases in aortic stiffness. Importantly, in young normotensive men, there is often no mid-systolic rise in aortic pressure (McEniery et al. 2005) and therefore AP is either zero or negative (Fig. 1A), unlike older normotensive men (Fig. 1B). Thus, in young men AP does not contribute to aSBP and therefore is not physiologically meaningful in the context of effecting increases in aSBP. Furthermore, inclusion of negative AP values can exaggerate relationships between variables (e.g. between AP and age), whereby this relationship does not exist following the removal of these values (Hughes et al. 2013). In the two aforementioned studies of young men, both negative and positive AP values were included in analyses (Casey et al. 2011; Smith et al. 2015); accordingly, the reported positive correlation between MSNA and AP may be exaggerated.

Herein, we aimed to (1) establish whether appropriate statistical adjustment influences the findings as to the effects of habitual endurance exercise on aortic haemodynamics, and, (2) assess the relationship between MSNA burst frequency and AP in normotensive young and middle-aged men. For aim 1, we hypothesised that adjusted aPWV and AP would not be different between groups due to training-mediated effects on heart rate and/or blood pressure, and that the age-related differences in aortic haemodynamics would be smaller in runners compared to nonrunners. To address aim 2, we merged the endurance-trained and recreationally active groups as MSNA burst frequency is not influenced by habitual exercise in either age group (Wakeham et al. 2019). Furthermore, we performed separate analyses for each age group due to higher MSNA and positive AP values in middle age (Matsukawa et al. 1998; McEniery et al. 2005). We hypothesised that there would be no significant correlation between MSNA and AP in either age group.

## Methodology

### Study overview

The individuals studied here have been reported on previously to address separate a priori study aims (Wakeham et al. 2019; Lord et al. 2020; Talbot et al. 2020). Forty-six healthy (free of chronic disease), non-smoking, normotensive and non-obese males were recruited into groups of endurance-trained runners and recreationally active nonrunners. Young runners had been training for 8 ± 5 years and were currently completing 65 ± 14 miles per week. The middle-aged runners had been training for 29 ± 15 years and were currently completing 35 ± 10 miles of per week. All endurance-trained runners were ranked within the top 30% of their age-category in the UK for 5 km in the year in which they were studied, with season best times of 15:22 ± 00:37 min:s and 19:31 ± 00:57 min:s for young and middle-aged runners, respectively. Nonrunners were recruited based upon self-report of completing at least 150 min of moderate to vigorous physical activity per week in accordance with the UK Physical Activity Guidelines for adults aged 19–64 years of age from the United Kingdom Chief Medical Officers (2019), but were not undertaking structured exercise training. All middle-aged men presented with a normal electrocardiogram at rest and during a maximal exercise test (via an incremental ramp protocol) on a cycle ergometer (Lode Corival, Groningen, Netherlands). Maximal exercise tests were used to determine cardiorespiratory fitness ($\dot{V}{\text{O}}_{2}$ Peak), via automated indirect calorimetry (Oxycon Pro, Jaeger, Hoechberg, Germany); the Oxycon Pro has been validated across a range of exercise intensities (Rietjens et al. 2001). This study conformed to the *Declaration of Helsinki*, other than registration in a database, and all procedures were approved by the Cardiff School of Sport and Health Sciences Research Ethics Committee (16/7/02R). Prior to testing, all participants provided both written and verbal informed consent.

### Study design

All visits were completed in a temperature-controlled laboratory (~ 22 °C). Participants attended the laboratory on two occasions having abstained from caffeine, alcohol, and strenuous exercise for twenty-four hours and fasted for six hours. The first visit involved the assessment of aortic haemodynamics, followed by the measurement of $\dot{V}{\text{O}}_{2}$ Peak. On a separate day, with a minimum of 1 week between testing days, participants underwent recordings of MSNA at rest, as outlined previously (Wakeham et al. 2019).

### Experimental measurements and data analyses

#### Aortic haemodynamics

The assessment of aortic haemodynamics was conducted as detailed previously (Talbot et al. 2020). Briefly, participants rested supine for 15 min prior to the assessment of brachial arterial blood pressure (Welch Allyn, UK) which was followed by the collection of radial arterial waveforms by applanation tonometry, using a high-fidelity micromanometer tipped probe (Millar Instruments), to estimate ascending aortic blood pressure (SphygmoCor, AtCor Medical, Australia). We determined the following parameters from the ascending aortic waveform, which was estimated using a generalised inverse transfer function (Pauca et al. 2001): P1 (first systolic shoulder of the aortic waveform; referred to as non-augmented aSBP), P2 (second systolic shoulder of the aortic waveform), AP (P2 – P1, mmHg), AIx ([AP/aortic pulse pressure] × 100, %), aSBP, aortic diastolic blood pressure (aDBP), aortic pulse pressure (aPP) and aortic mean arterial pressure (aMAP; Fig. 1). AP and AIx were also extracted following adjustment for a heart rate of 75 bpm (AP@75 and AIx@75, respectively) within the SphgymoCor software, which is calculated based upon the regression equations from Wilkinson and colleagues (2000; 2002), as detailed previously (Stoner et al. 2014). We also quantified pulse pressure amplification (brachial PP-aPP). All data were collected in triplicate in accordance with the quality criteria within the SphygmoCor software by one trained observer (TGD), with an average reported herein. AP is the primary index of aortic systolic pressure augmentation in this study as AP increases linearly with age (McEniery et al. 2005) and is not influenced mathematically by the concurrent increase in aPP, unlike AIx (Namasivayam et al. 2010). We consider AP as an index of aortic systolic pressure augmentation only, not aortic reservoir pressure nor the magnitude of backward travelling pressure waves per se (Hughes et al. 2013).

Aortic (aPWV) and brachial pulse wave velocity (bPWV) were then determined in accordance with current guidelines (Townsend et al. 2015), by dividing the transit time between the foot of the carotid and femoral, or radial, arterial waveforms, respectively, by the path length. To enable comparison of data with the published reference values (Reference Values for Arterial Stiffness, 2010), raw aPWV was converted using the following equation: converted aPWV = (raw carotid-femoral distance × 1.12) ÷ time (Van Bortel et al. 2012; Huybrechts et al. 2011). Both raw and converted aPWV are reported in line with recent suggestions (Van Bortel et al. 2020). We also quantified the arterial stiffness gradient as the ratio of raw bPWV and converted aPWV (Niiranen et al. 2017).

Raw AP and aPWV are influenced by heart rate, (a) MAP, age and height (for AP only) (Stoner et al. 2014; Tan et al. 2016; Van Bortel et al. 2020). In the presence of significant differences in these covariates, AP and aPWV are also presented in adjusted form (via analysis of covariance [ANCOVA]), to determine the effects of habitual exercise and age on *intrinsic* aortic stiffness (aPWV) and associated haemodynamics (AP). If converted aPWV differs between groups there would be a functionally relevant difference in *operating* aortic stiffness, as this measure reflects windkessel function which will contribute to haemodynamic regulation. However, if adjusted aPWV is not different between groups this would suggest that there is no difference in *intrinsic* aortic stiffness (i.e. aPWV independent of confounders) and that the difference in *operating* aortic stiffness is mediated by the factors adjusted for. This is similar for AP; whereby, if measured AP differs between groups this additional haemodynamic load if faced by the left ventricle; however, if this difference does not persist for adjusted AP it highlights that this difference is mediated by the factors adjusted for. As age is an independent variable in this study, age was not included as a covariate. Raw bPWV was also statistically adjusted (via separate ANCOVAs) for MAP and heart rate. The adjustment for aPWV and bPWV for aMAP and MAP, respectively, was due to the significant difference between aMAP and MAP in young but not middle-aged men (*Young*: mean difference [95% confidence interval], − 4 [− 6 to − 2] mmHg, *P* < 0.001; *Middle-aged*: − 1 [− 2 to 1] mmHg, *P* = 0.422; via independent-samples *t* test).

#### Muscle sympathetic nerve activity

Recordings of multi-unit MSNA were taken from the peroneal nerve using microneurography, as previously described (Wakeham et al. 2019). In brief, a tungsten microelectrode was inserted into the peroneal nerve, by one trained microneurographer (JPM), with a reference electrode inserted 2–3 cm away to obtain the raw nerve signal which was amplified, filtered, rectified and integrated (time constant 0.1 s; Nerve Traffic Analyser, Model 663 C, Iowa). MSNA was sampled at 1 kHz onto a personal computer using a commercially available data acquisition system (Chart Version 8, LabChart Pro, ADInstruments, UK) and saved for offline analysis. MSNA was analysed as previously described (Wakeham et al. 2019) and is presented as burst frequency (bursts min^−1^) and burst incidence (bursts 100 hb^−1^) for the assessment of between-group differences. Notably, MSNA burst frequency is the primary MSNA variable of interest for the correlational analyses, as it reflects the temporal pattern of sympathetic outflow to the vasculature which determines neurotransmitter release; whereas, MSNA burst incidence reflects the arterial baroreflex control of MSNA (Charkoudian and Wallin 2014).

### Statistical analyses

To address the first aim of this study, after confirming compliance with basic parametric assumptions, analysis of variance (ANOVA) was used to compare participant characteristics. Subsequently, a general linear model was used to compare aortic haemodynamics between groups; that is to determine the main effects of runner and age, and the runner*age interaction. In the presence of a significant runner*age interaction, SIDAK post-hoc multiple comparisons were assessed. In the presence of significant main effects of runner or age for Converted aPWV or AP, as well as for the potential covariates (heart rate and aMAP [or height, for AP only]), then ANCOVA analyses were performed. The statistical model used for the ANCOVA was dependent on the presence of a significant runner*age interaction. If there was no significant runner*age interaction, then a general linear model was used with the inclusion of the significant covariates; however, if there was a significant interaction, separate one-way “posthoc” ANCOVA analyses were conducted for each of the four primary between-group comparisons (i.e. effects of runner in young [1] and middle-aged men [2], as well as the effects of age in nonrunners [3] and runners [4]). Adjusted group standard deviations (SD) were calculated from the sum of the square root of the sample size and the standard error of the estimated marginal means from the adjusted statistical models (Atkinson and Batterham 2013). Mean difference [95% confidence intervals] and Hedges’* g*, an estimate of effect size ([−]0.2 small effect, [−]0.5 medium effect, [−]0.8 large effect) (Cohen 1992), are also reported for primary outcomes, namely adjusted aPWV, adjusted AP, and aSBP. To address the second aim of this study, two-tailed Pearson product-moment correlation coefficients were generated to assess the relationship between MSNA and AP for the merged samples of young (*n* = 23) and middle-aged (*n* = 23) men. Data are presented as mean ± standard deviation or as mean difference [95% confidence intervals] and α was set a priori at < 0.05. All statistical analyses were conducted using Statistics Package for Social Sciences (SPSS) for Windows (version 26, Chicago, IL).

## Results

### Participant characteristics

In both young and middle-aged men, body mass and BMI were significantly lower in runners compared to nonrunners, whereas $\dot{V}{\text{O}}_{2}$ Peak was higher in runners (Table 1). $\dot{V}{\text{O}}_{2}$ Peak was lower in middle-aged compared to young runners, with no difference between middle-aged and young nonrunners.

**Table 1:** Participant characteristics

Variable	Young nonrunners	Young runners	Middle-aged nonrunners	Middle-aged runners	*P*
*n*	10	13	10	13	
Demographics					
Age (years)	23 ± 3	22 ± 3	53 ± 2^‡^	57 ± 5^‡^	** < 0.001**
Stature (cm)	178.1 ± 5.8	179.9 ± 5.1	175.6 ± 7.1	174.7 ± 6.3	0.139
Body mass (kg)	80.4 ± 16.2	67.0 ± 5.1^†^	80.9 ± 9.9	66.1 ± 7.9^†^	** < 0.001**
BMI (kg m^2^)	25.4 ± 4.6	20.8 ± 1.3^†^	26.2 ± 3.1	21.6 ± 1.6^†^	** < 0.001**
Cardiorespiratory fitness					
$\dot{V}{\text{O}}_{2}$ Peak (mL min^−1^)	2894 ± 508	4018 ± 607 †	2641 ± 726	3349 ± 572^†‡^	** < 0.001**
$\dot{V}{\text{O}}_{2}$ Peak (mL kg^−1^ min^−1^)	36.5 ± 6.3	60.6 ± 9.3^†^	32.6 ± 8.4	50.7 ± 6.1^†‡^	** < 0.001**

Data are presented as mean ± standard deviation. Data were compared via ANOVA with SIDAK post hoc multiple comparisons when appropriate (i.e. *P* < 0.05 from ANOVA). *P* values in bold are considered statistically significant

*BMI *body mass index, $\dot{V}O_{2}$ volume of oxygen

^†^Represents *P* < 0.05 compared to age-matched non-runner

^‡^Represents *P* < 0.05 compared to young counterparts

### Study aim 1: effects of habitual exercise and age on aortic haemodynamics

#### Blood pressure

**Fig. 2:**
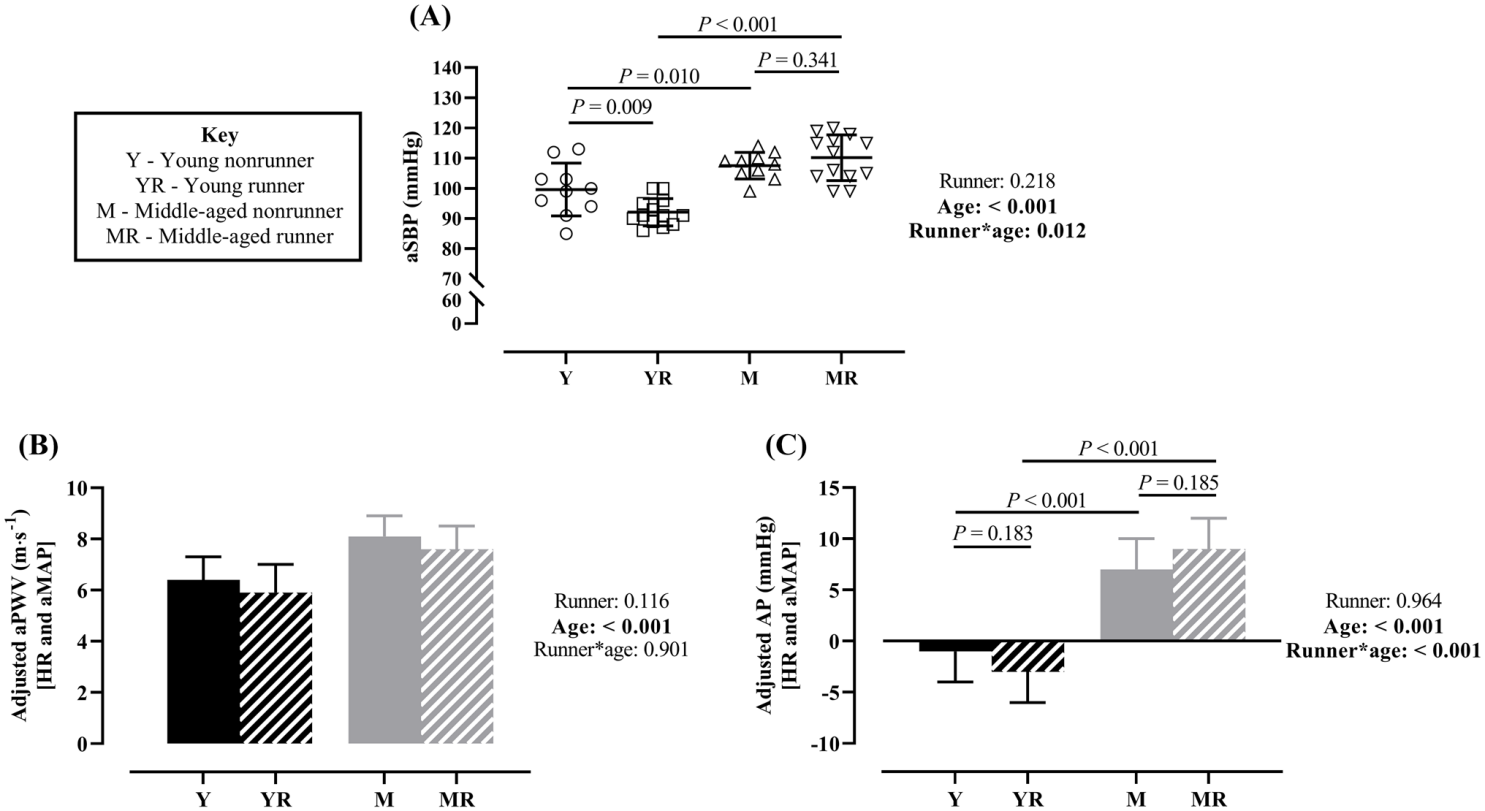
Effects of habitual exercise and age on aortic haemodynamics. **A** There was a significant runner*age interaction for aSBP. Specifically, aSBP was lower in young runners compared to nonrunners, with no difference between middle-aged groups; thus, the age-related difference was greater in runners compared to nonrunners (see text). **B** Age, but not habitual endurance exercise, increased adjusted aPWV; whereby aortic stiffness was higher with age in both runners and nonrunners. **C** There was no effect of habitual exercise on adjusted AP; however, age was associated with higher systolic pressure augmentation. As detailed within text, the magnitude of difference with age was greater in runners compared to nonrunners. NB: All data are presented with standard deviation error bars. The main effects and interaction, with SIDAK post hoc tests of multiple comparisons are presented from a general linear model for aSBP (**A**). Covariates, noted in [parentheses], were added to the general linear model to determine between-group differences in Adjusted aPWV (**B**) and AP (**C**). Separate ANCOVA analyses were conducted to determine between-group differences in Adjusted AP due to the significant runner*age interaction (**C**). *AP *augmentation pressure,* aPWV *aortic pulse wave velocity,* aMAP *aortic mean arterial pressure,* aSBP *aortic systolic blood pressure,* HR *heart rate

Heart rate, non-augmented aSBP, aMAP, DBP and MAP were lower in runners compared to nonrunners (main effect of runner); whereas, non-augmented aSBP, aSBP, aDBP, aPP, aMAP, DBP, MAP and PPA were higher with age (main effect of age; Table 2). There was a significant runner*age interaction for aSBP (Fig. 2A), whereby aSBP was lower in young runners compared to nonrunners (− 8 [− 13 to − 3] mmHg, Hedges’* g* = 1.1) with no difference between middle-aged runners and nonrunners (3 [− 3 to 8] mmHg, Hedges’* g* = − 0.3). Thus, there was a larger mean difference in aSBP with age in runners (18 [13 to 23] mmHg, Hedges’* g* = 2.6) compared to nonrunners (8 [2–14] mmHg, Hedges’* g* = 1.1).

**Table 2:** Haemodynamics

	Young nonrunners	Young runners	Middle-aged nonrunners	Middle-aged runners	*P* values		
Variable					Runner	Age	Runner*Age
*n*	10	13	10	13			
Arterial stiffness							
Raw aPWV (m s^−1^)	5.8 ± 0.7	5.1 ± 0.5	7.5 ± 0.8	6.8 ± 0.9	**0.002**	** < 0.001**	0.992
Converted aPWV (m s^−1^)	6.5 ± 0.8	5.7 ± 0.5	8.4 ± 1.0	7.6 ± 1.0	**0.002**	** < 0.001**	0.998
Raw bPWV (m s^−1^)	7.0 ± 0.9	6.2 ± 0.4	7.3 ± 0.7	7.2 ± 0.6	**0.030**	**0.001**	0.091
Adjusted bPWV (m s^−1^)	7.0 ± 0.8	6.1 ± 0.8	7.4 ± 0.7	7.2 ± 0.8	**0.021**	** < 0.001**	0.080
bPWV/aPWV	1.2 ± 0.2	1.2 ± 0.2	1.0 ± 0.1	1.1 ± 0.1	0.199	** < 0.001**	0.348
Systolic pressure augmentation							
Raw AP (mmHg)	– 2 ± 2	– 3 ± 2	6 ± 3^‡^	10 ± 3^†‡^	**0.039**	** < 0.001**	**0.001**
AP@75 (mmHg)	– 4 ± 3	– 7 ± 3^†^	3 ± 3^‡^	4 ± 2^‡^	0.165	** < 0.001**	**0.013**
Raw AIx (%)	– 6 ± 9	– 11 ± 9	19 ± 9^‡^	28 ± 8^†‡^	0.450	** < 0.001**	**0.008**
AIx@75 (%)	– 13 ± 8	– 26 ± 9^†^	10 ± 8^‡^	12 ± 5^‡^	**0.036**	** < 0.001**	**0.004**
Heart rate and blood pressure							
Heart Rate (bpm)	60 ± 12	46 ± 7	57 ± 11	43 ± 9	** < 0.001**	0.291	0.949
Non-augmented aSBP (mmHg)	99 ± 9	92 ± 4	102 ± 6	100 ± 7	**0.040**	**0.011**	0.175
aDBP (mmHg)	73 ± 10	66 ± 6	78 ± 5	76 ± 9	0.071	**0.003**	0.264
aPP (mmHg)	27 ± 8	26 ± 4	30 ± 4	34 ± 6	0.257	**0.002**	0.142
aMAP (mmHg)	82 ± 5	78 ± 6	90 ± 5	88 ± 7	**0.046**	** < 0.001**	0.646
SBP (mmHg)	119 ± 13	111 ± 5	119 ± 7	118 ± 8	0.114	0.218	0.180
DBP (mmHg)	71 ± 7	66 ± 6	76 ± 5	74 ± 7	**0.030**	** < 0.001**	0.322
PP (mmHg)	47 ± 11	45 ± 7	42 ± 6	44 ± 5	0.936	0.109	0.418
MAP (mmHg)	87 ± 8	81 ± 5	90 ± 5	89 ± 7	**0.035**	**0.005**	0.215
PPA (mmHg)	21 ± 7	19 ± 3	12 ± 4	9 ± 7	0.215	** < 0.001**	0.677

Data are mean ± SD. Annotated data points represent significance from post-hoc multiple comparisons following ANOVA and ANCOVA for raw and adjusted data, respectively. *P* values in bold are considered statistically significant

*a *aortic,* AIx *augmentation index,* AP *augmentation pressure,* b *brachial,* DBP *diastolic blood pressure.* MAP *mean arterial pressure,* PP *pulse pressure,* PPA *pulse pressure amplification,* PWV *pulse wave velocity,* SBP *systolic blood pressure

^†^Represents *P* < 0.05 compared to age-matched non-runner

^‡^Represents *P* < 0.05 compared to young counterparts

Significant main effects of runner and age or significant runner*age interactions are in bold

#### Arterial stiffness

Converted aPWV (i.e. unadjusted) was lower in runners compared to nonrunners and higher in middle-aged compared to young men (Table 2). However, adjusted aPWV was not different between young (− 0.5 [− 1.2 to 0.3] m s^−1^, Hedges’* g* = -0.5) or middle-aged (− 0.5 [− 1.3 to 0.3] m s^−1^, Hedges’* g* = -0.6) runners and nonrunners, but was higher in middle-aged men (nonrunners: 1.7 [0.9–2.5] m s^−1^, Hedges’* g* = 1.6; runners: 1.7 [0.9–2.4] m s^−1^, Hedges’* g* = 1.9); there was no significant runner*age interaction (Fig. 2B). Results were similar for raw and adjusted bPWV; whereby, bPWV was lower in runners compared to nonrunners and higher with age, with no runner*age interaction. The arterial stiffness gradient (bPWV-aPWV) was higher with age only (Table 2).

#### Systolic pressure augmentation

There was a significant runner*age interaction for raw AP. Specifically, raw AP was higher in middle-aged runners compared to nonrunners (*P* < 0.001), with no significant difference between young groups (*P* = 0.360); thus, the age-related difference was greater in runners (13 [11–15] mmHg, *P* < 0.001) compared to nonrunners (8 [5–10] mmHg, *P* < 0.001). When adjusted via ANCOVA, AP (Fig. 2C) was not different between runners and nonrunners in either the young (− 2 [− 4 to 1] mmHg, Hedges’* g* = − 0.6) or middle-aged groups (2 [− 1 to 4] mmHg; Hedges’* g* = 0.6), but was higher with age in nonrunners (8 [5–11] mmHg, Hedges’* g* = 2.6) and runners (11 [8–13] mmHg, Hedges’* g* = 3.6), with a slightly larger effect in runners, similar to the finding for raw AP. Notably, these results differ as to the effects of habitual exercise when using either AP@75 (or AIx@75); whereby, AP@75 is lower in young runners compared to nonrunners (*P* < 0.001) but not different between middle-aged runners and nonrunners (*P* = 0.420).

### Study aim 2: relationships between MSNA and AP

When the runners and nonrunners were merged within each age group to address the second study aim, middle-aged men were older (55 ± 4 vs 23 ± 3 years, *P* < 0.001) and shorter (175.1 ± 6.5 vs 179.1 ± 5.4 cm, *P* = 0.025) than young men. Body mass (72.6 ± 11.4 vs 72.8 ± 12.9 kg, *P* = 0.946), BMI (24 ± 3 vs 23 ± 4 kg m^2^, *P* = 0.433) and $\dot{V}{\text{O}}_{2}$ Peak (43.8 ± 10.9 vs 50.1 ± 14.6 mL kg^−1^ min^−1^, *P* = 0.067) were not different between middle-aged and young men. MSNA burst frequency (30 ± 10 vs 17 ± 8 bursts min^−1^, *P* < 0.001) and incidence (62 ± 21 vs 32 ± 18 bursts 100hb^−1^, *P* < 0.001) were higher in middle-aged men.

**Fig. 3:**
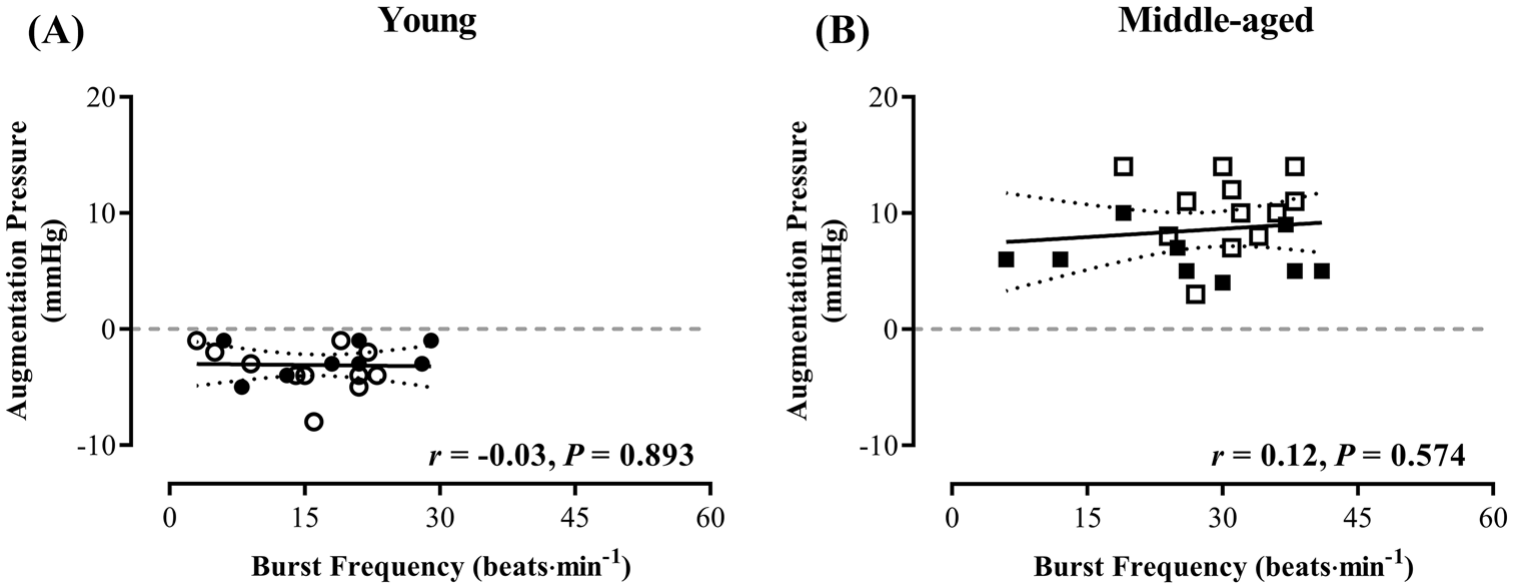
The relationship between muscle sympathetic nerve activity and aortic augmentation pressure. There were no significant correlations between muscle sympathetic nerve activity (MSNA) burst frequency and augmentation pressure in young (**A**; *n* = 19) or middle-aged (**B**; *n* = 21) normotensive men. 5 participants were excluded from these analyses (4 young [2 runners] and 1 middle-aged nonrunner) due to AP values ≥ 0 in the young group or ≤ 0 in the middle-aged group. NB: Dotted lines represent the 95% confidence intervals. Open symbols represent the runners within each age group

As 4 young men had AP ≥ 0 and 1 middle-aged man had an AP of 0, separate analyses for positive and negative AP values was not possible in each age group. Correlation analyses were, therefore, conducted in 19 young men and 22 middle-aged men, with only negative and positive AP values, respectively. We observed no significant relationship between MSNA burst frequency and AP in either young or middle-aged men (Fig. 3). In addition, there was no significant correlation between MSNA and AIx in either young (*r* = − 0.03, *P* = 0.893) or middle-aged (*r* = 0.12, *P* = 0.574) men.

## Discussion

The principal findings from this study are as follows. First, the statistical adjustment of aPWV and AP for aMAP and heart rate changed the conclusions as to the effects of habitual endurance; adjusted aPWV and AP were not different between trained and untrained men, which contrasts the lower unadjusted values of Converted aPWV and AP. Second, there was an age-dependent effect of habitual exercise on aSBP, whereby aSBP was lower in young runners compared to nonrunners only. Accordingly, the magnitude of difference in aSBP was greater with age in runners compared to nonrunners, with a similar age-related difference for aPWV and AP. Third, we report no significant relationship between MSNA burst frequency, in other words sympathetic vasomotor outflow, and AP in young or middle-aged men, with negative and positive AP, respectively. These data suggest that habitual exercise is associated with a different aortic haemodynamic profile in young compared to middle-aged men and habitual endurance exercise does not influence aortic stiffness or augmentation pressure when adjusted for the physiological effects of heart rate and blood pressure. Furthermore, the data presented here show that aortic systolic pressure augmentation does not correlate with the level of sympathetic vasoconstrictor drive to skeletal muscle in groups of active young or middle-aged normotensive men.

### Effects of habitual exercise on aortic haemodynamics in young and middle-aged men

In the present study, HR and aMAP adjusted aPWV was not influenced by habitual exercise in either young or middle-aged men. In the studies conducted to date aPWV has not been adjusted for HR and aMAP (Vaitkevicius et al. 1993; Tanaka et al. 1998; McDonnell et al. 2013; Pierce et al. 2013, 2016; Tarumi et al. 2013, 2021). Notably, the adjustment of aPWV markedly changed our study findings. Converted aPWV (i.e. unadjusted) was lower with habitual exercise in both young and middle-aged men, as reported in most (Vaitkevicius et al. 1993; Tanaka et al. 1998; McDonnell et al. 2013; Pierce et al. 2013, 2016; Tarumi et al. 2013, 2021; Otsuki et al. 2007a, b), but not all (Bjarnegard et al. 2018; McDonnell et al. 2013; Shibata et al. 2018) previous studies. Our findings suggest that habitual exercise may not directly influence *intrinsic* aortic stiffness (i.e. aPWV independent of confounders) per se, rather that habitual exercise influences *operating* aortic stiffness (i.e. measured aPWV) due to training-related effects on heart rate and arterial pressure. Thus, future studies should also determine adjusted aPWV when appropriate, dependent on differences in heart rate and/or arterial pressure, to avoid reporting erroneous conclusions (Van Bortel et al. 2020) regarding the effects of habitual exercise on *intrinsic* aortic stiffness. These data suggest that habitual exercise does not change *intrinsic* aortic stiffness in healthy normotensive men.

Alongside aortic stiffness, aortic systolic pressure augmentation is an important determinant of aortic blood pressure, which independently increases cardiovascular risk (Wang et al. 2010). When adjusted for heart rate and aMAP, there were no significant effects of habitual exercise on AP (or AIx) in young or middle-aged men. Again, these findings differ when these variables are unadjusted, as AP was not different in young men, but AP was higher in middle-aged runners compared to nonrunners. AP@75 was, however, lower in young runners compared to nonrunners, whereas the normalisation to a heart rate of 75 bpm removed the difference between middle-aged groups. Thus, the adjustment of AP for heart rate and aMAP highlights the importance of including aMAP as a covariate when comparing AP between the trained and untrained men here, as adjusted AP was not effected by habitual exercise in either age group. The use of ANCOVA for the adjustment of AP or AIx for heart rate, as opposed to the automated adjusted to 75 bpm within the SphygmoCor, is beneficial as this statistical method adjusts the dependent variable according to the group means and not based upon an arbitrary calculation from previous data, as highlighted previously (Stoner et al. 2014). Furthermore, the adjustment to 75 bpm is limited to heart rates between 40 and 110; thus, it is not possible to generate these data in endurance athletes who present with heart rates below 40 bpm, as was the case for four runners included here. Previous studies have reported that systolic pressure augmentation (AP, AIx or AIx@75) is either lower (Edwards and Lang 2005; Denham et al. 2016; McDonnell et al. 2013; Bjarnegard et al. 2018) or not different (Knez et al. 2008; McDonnell et al. 2013; Shibata et al. 2018) with habitual exercise. The mechanism(s) mediating the higher raw AP in middle-aged runners compared to nonrunners are unclear; future studies are required to determine the influence of habitual exercise across the lifespan on forward, backward and reservoir pressures. The disparity in findings is likely due to the differences in the measure (AP vs AIx), or equipment used, whether, or not, heart rate and aMAP were adjusted for, as well as the magnitude of differences in cardiorespiratory fitness and/or participant age. Together, the data presented here suggest that the habitual endurance exercise related difference in aortic systolic pressure augmentation is mediated by heart rate and aMAP.

The effects of habitual exercise on aSBP were age-dependent; whereby, aSBP was only lower in young runners compared to nonrunners, despite no significant differences in adjusted aPWV or AP in either age group. This age-dependent effect is likely due to the greater magnitude of difference in non-augmented aSBP (i.e. P1) in young runners compared to nonrunners (-6 mmHg); the mean difference was smaller (− 2 mmHg) between middle-aged groups. As P1 is determined by the interaction between the left ventricular ejection wave and ascending aortic stiffness, the greater ascending aortic compliance in young endurance-trained men (Tarumi et al. 2021) likely contributes to this difference in non-augmented aSBP. The effects of habitual exercise on ascending aortic stiffness in middle/older age are currently unclear. It is important to note that our data do contradict a previous study which reported the inverse, with effects of habitual exercise on aSBP in middle-aged but not young individuals (McDonnell et al. 2013). However, this discrepancy is likely related to the study of only normotensive men in both age groups here, unlike the previous study. Therefore, in terms of cardiovascular risk as determined by aSBP (Stamatelopoulos et al. 2006; Vlachopoulos et al. 2010; Lamarche et al. 2020), the benefit of habitual endurance exercise is apparent in young but not middle-aged normotensive men.

### Effect of age in runners and nonrunners

With advancing age aortic stiffness, systolic pressure augmentation, and aortic blood pressure increase (McEniery et al. 2005). Many studies have suggested that lifelong habitual exercise can slow age-related aortic stiffening (Vaitkevicius et al. 1993; Tanaka et al. 1998; Gates et al. 2003; Pierce et al. 2013, 2016; McDonnell et al. 2013), which could also influence AP. However, some of these previous studies compared middle/older aged endurance-trained individuals with young sedentary, but not young endurance-trained, individuals (Vaitkevicius et al. 1993; Pierce et al. 2013, 2016), which could underestimate the age-related difference with the middle/older aged endurance-trained groups. Indeed, we report that the age-related differences in aPWV are similar for chronically endurance-trained runners and recreationally-active nonrunners. Importantly, the middle-aged runners here had been training for ~ 30 years, which is similar to the age difference between our young and middle-aged groups (~ 32 years). This finding opposes the view that habitual endurance exercise has “anti-ageing” effects on aortic stiffness, as reviewed recently (Nowak et al. 2018; Rossman et al. 2018; Seals et al. 2018; Tanaka 2019; Seals et al. 2019; Parry-Williams and Sharma 2020). The age-related difference in AP was greater (larger effect size) in runners compared to nonrunners. This also contradicts a previous report of no effect of habitual exercise on the age-related difference in AP has been reported previously (McDonnell et al. 2013); however, as mentioned previously, the older groups studied by McDonnell and colleagues were prehypertensive and hypertensive, unlike the present study, and the active group were likely not as homogenous as the sample studied here. Thus, more studies are warranted to provide further insight into whether habitual endurance exercise influences “aortic ageing”, utilising a similar four-group study design as utilised here, with appropriate adjustments of aPWV and AP.

In the present study, the age-related difference in aSBP was greater in runners compared to nonrunners. Despite this, the middle-aged runners did not present with higher aSBP or adjusted AP when compared to their recreationally active age-matched counterparts (Table 2). This is of importance for their cardiovascular risk profile, as absolute aSBP is predictive of cardiovascular morbidity and mortality (Stamatelopoulos et al. 2006; Vlachopoulos et al. 2010; Lamarche et al. 2020).

### Relationship between MSNA and AP

In the current study, we found no relationship between MSNA burst frequency and AP, in young or middle-aged normotensive men. Specifically, we observed this in a sample of young men where AP did not contribute to aSBP in 91% of individuals, but did contribute to aSBP in 96% of middle-aged men, due to the return of the backward pressure wave occurring in diastole and systole in young and middle-aged men, respectively. MSNA and AP (or AIx) have been shown to positively correlate in young men (Casey et al. 2011; Smith et al. 2015); however, 44% (Casey et al. 2011) and 53% (Smith et al. 2015) of the samples studied previously had positive AP values. Whether these previously reported correlations between MSNA and AP in young men would remain when assessed for positive and negative AP values separately, is unclear. The previous studies in middle/older age report disparate findings. Millar et al. (2019) reported no correlation between MSNA and AP in healthy normotensive individuals (75% male), where AP contributed to aSBP in 88% of participants studied. The discordance in findings between this study and those conducted previously likely reflects differences in the distribution of AP values and/or levels of aortic pressure. Indeed, higher arterial pressure is associated with higher AP (Mitchell 2014). Positive relationships between MSNA and AP may only exist in individuals with elevated arterial pressure and AP, as shown previously in prehypertensive and hypertensive postmenopausal women (Hart et al. 2013) and heart failure patients with reduced ejection fraction (Millar et al. 2019). Together, in normotensive young or middle-aged men, without and with pressure raising AP values respectively, resting peripheral sympathetic vasomotor outflow does not correlate with AP (or AIx).

Casey et al. (2011) suggested that MSNA likely contributes to AP in young men via alterations in peripheral vascular tone, and subsequent increases in backward travelling pressure waves, as well as increases in aortic stiffness (Casey et al. 2011). However, the authors made this conclusion despite reporting no significant correlation between total peripheral resistance and AP in young men (Casey et al. 2011). Regarding the influence of MSNA on aortic stiffness, and subsequently AP, it has been shown that MSNA significantly correlates with aPWV at rest (Swierblewska et al. 2010; Holwerda et al. 2019) and there are pressure-independent increases in aPWV during acute sympathoexcitation (Nardone et al. 2018; Holwerda et al. 2019). Notably, we observe no significant correlation between MSNA and aPWV in either age group studied here (young: *r* = 0.162, *P* = 0.461; middle-aged: *r* = − 0.367, *P* = 0.085), which may contribute to the lack of relationships between MSNA and AP in this study. Thus, a positive relationship between MSNA and aortic stiffness may underlie the previously reported positive correlations between MSNA and AP (Casey et al. 2011; Smith et al. 2015; Hart et al. 2013; Millar et al. 2019); an effect likely due to the pressure-raising effect higher aortic stiffness has upon aortic reservoir pressure, and subsequently AP (Davies et al. 2010). This may especially be the case in the presence of high blood pressure and/or cardiovascular disease where AP raises aSBP (Mitchell 2014).

### Methodological considerations

There are some limitations which warrant discussion. First, as we only studied Caucasian men here it is not possible to determine if there are similar or divergent effects of habitual exercise and/or ageing on aortic haemodynamics between the sexes or different racial groups. The effects of habitual exercise on central haemodynamics have been studied in women previous, with reports of no differences between trained and untrained women in either young (Bjarnegard et al. 2018) or middle/older age (Tanaka et al. 1998). Further, there are no effects of sex on aortic pressure in either African American or Caucasian individuals, despite lower aPWV in women (Yan et al. 2014). Future studies are required to understand the interaction between the effects of habitual exercise, age, sex, and other socio-cultural factors on aortic haemodynamics. Second, as a cross-sectional study design was utilised, it is not possible to truly determine the effects of habitual exercise on the age-related changes in aortic haemodynamics. Future studies should complete longitudinal follow-up studies in endurance-trained and untrained men and women to discern the effects of age, sex and habitual exercise on aortic haemodynamics. Furthermore, as we did not record objective measures of physical activity here, we cannot determine the effects of different physical activity and/or sedentary time on aortic haemodynamics. This was by design, as we studied healthy but untrained men who were meeting the current physical activity guidelines. Third, we assessed the relationships between resting MSNA and aortic AP which were recorded on separate days. However, the measurement of MSNA is repeatable within individuals over the short, medium and long-term (Fagius and Wallin 1993; Kimmerly et al. 2004; Notay et al. 2016; Grassi et al. 1997; Fonkoue and Carter 2015). Finally, it is not possible to directly record sympathetic outflow to the aorta in humans. Nevertheless, the main aim of the correlational analyses performed here was to determine whether peripheral sympathetic vasomotor outflow (i.e. MSNA) is correlated to AP with consideration of the effect of negative and positive AP values.

### Conclusion

We report an age-dependent effect of habitual endurance exercise on aortic haemodynamics in normotensive men; whereby, aSBP is lower in young, but not middle-aged, runners compared to nonrunners, with no difference in adjusted aPWV or AP in either age-group. The difference in findings between the raw and adjusted aPWV and AP data highlight that the appropriate adjustments of aortic stiffness and systolic pressure augmentation are important when studying the effects of habitual exercise between groups of young and middle-aged men to avoid reporting erroneous conclusions (Van Bortel et al. 2020). However, the differences in unadjusted aPWV and AP with habitual exercise reported here are the functionally relevant *operating* aortic stiffness and augmentation pressure values, which are of clear physiological and potential clinical relevance. Nevertheless, our study highlights that *intrinsic* aortic stiffness and adjusted systolic augmentation pressure are not influenced by habitual exercise. Thus, the habitual exercise related differences in *operating* aortic stiffness and systolic augmentation pressure appear to be mediated by differences in other hemodynamic indices, namely heart rate and blood pressure. In addition, we report no significant correlation between peripheral sympathetic vasomotor outflow and systolic pressure augmentation in either young or middle-aged normotensive men. Future studies should consider performing separate correlational analyses with positive and negative AP values, as only positive AP values represent clinically relevant physiological effects on aSBP.

## Data Availability

Data are available from the corresponding author upon reasonable request.
